# Channelling and taxation in European online gambling markets: evolution and policy implications

**DOI:** 10.1186/s12954-024-01145-0

**Published:** 2025-01-03

**Authors:** Virve Marionneau, Nicola Matteucci, Sabrina Vieira Lima, Janne Nikkinen, Jani Selin

**Affiliations:** 1 Centre for Research on Addiction, Control, and Governance, Faculty of Social Sciences. Unioninkatu 33, University of Helsinki, 00014 Helsinki, Finland; 2 DiSES, Marche Polytechnic University, P.le Martelli, 8, 60121 Ancona, Italy; 3 Independent Researcher, Ancona, Italy; 4 Finnish Institute for Health and Welfare, Mannerheimintie 166, 00271 Helsinki, Finland

**Keywords:** Gambling, Taxation, Channelling, Offshore, Regulation, Harm reduction, Europe

## Abstract

**Background:**

Taxation can be used to direct consumption and provision of harmful commodities. Prior research on gambling taxation has nevertheless been inconclusive on whether this can also apply to gambling. In gambling policy, optimal taxation rates have particularly been debated from the perspective of channelling consumption from offshore markets to regulated markets. Prior industry-sponsored reports have suggested that lower tax rates may be correlated with higher channelling rates.

**Methods:**

We analyse data on two cross-sections (2018; 2021) derived from 29 European countries. The data consist of estimated channelling rates, information on taxation levels, and controls including blocking policies. We produce a descriptive overview of the recent evolution of market channelling and taxation for online gambling products across Europe. We also produce a multivariate regression analysis on the extent that market channelling is correlated with taxation of online gambling.

**Results:**

Our results show important divergence in taxation of online gambling markets in Europe. We also found that over time, the market share of offshore markets has declined in relative terms. However, this decline is explained by a more rapid growth in the regulated market in absolute terms. The regression analysis found no evidence of a negative correlation between that taxation rates and channelling rates within Europe.

**Conclusions:**

Gambling policy needs to be based on empirical, impartial evidence. Misleading estimates may result in increased harms to societies. Channelling objectives are important for better regulation and harm reduction, but taxation levels do not appear to be correlated to the success of channelling policies.

**Supplementary Information:**

The online version contains supplementary material available at 10.1186/s12954-024-01145-0.

## Background

Taxation is used to direct consumption of many harmful commodities. For example, excise taxation has been used as a policy measure to reduce the consumption of alcohol, tobacco, or high-sugar beverages [7, 8, 18, 51]. Gambling is also traditionally taxed at rates above those of many other sectors. Taxation has been expected to direct consumption and to produce revenue for governments [27, 47], although the effects of taxation on directing gambling consumption are likely less straightforward than for many other commodities. Financial losses are a central factor in producing gambling harm and addiction, and consumption is highly concentrated [5, 36, 42, 52, 54]. The elasticity of demand for gambling may be inelastic, particularly amongst those who experience gambling-related problems [9, 23, 48, 49, 57]. Pricing can therefore have an effect on some consumers, but not necessarily those experiencing the most harm [49]. Furthermore, gambling products are designed in a manner that obfuscates the actual price of participation [62].

### Prior research on gambling taxation

Jurisdictions differ significantly in how they tax gambling (e.g., [22, 47]). Comparisons across countries are further complicated by differences in mechanisms through which surplus from gambling is collected to states. Gambling taxation can take place at various levels: states can tax gross gambling revenue (GGR), turnover (R), or impose unit taxes for gambling devices and servers. In some countries, gambling winnings are subject to income tax. Gambling can also take place either at point-of-consumption or point-of-sale basis. In addition to taxation, gambling can produce surplus to states via license fees, state ownership and dividends, or arrangements by which gambling profits are transferred directly to ‘good causes’ within the state or the third sector [10, 17, 39].

Studies comparing gambling tax incidence across jurisdictions have been scarce. An early study comparing gambling tax rates in Europe [22] found that across countries, lottery taxation varied from 12 to 50 percent/GGR. Taxation on casino products varied from 20 to 92 percent/GGR. These comparisons were complicated by the prevalence of state monopolies. Another study comparing tax rates in European countries [34] reported an average tax rate of 27 percent/GGR for land-based gambling (N = 13), but only 19 percent/GGR for online gambling (N = 15). A study from the United States using data from 1996 [10] similarly found that tax rates can vary significantly across products. Overall, table games at US casino jurisdictions were taxed at only about 2 percent/GGR, while state lotteries were taxed on average at 40 percent/GGR.

Some research has addressed the effects of tax mechanisms and rates on markets and consumers. One study [58] compared different forms of betting (fixed odds, spread bets and pari-mutuel betting), showing that taxation on volume impacts odds and commissions, but a similar effect was not observed when taxation was based on profit. There have also been reports on the effects of growing gambling markets to state coffers or other beneficiaries, and associated state interests in maintaining or even increasing this revenue [39, 41, 55, 60].

Other research has focused on the consumer experience and how taxation can impact consumer surplus [22, 49]. There is evidence that the taxation of gambling can only direct the consumption of some consumer segments [9, 49]. Fiedler [24] has argued that this inelasticity partly supports higher taxation to reduce demand.

Finally, a body of research has focused on tax incidence in societies, and particularly the highly regressive nature of gambling taxation [2, 12, 26, 31, 53].

### Taxation and channelling

From the perspective of the gambling industry or regulators, taxation can also be seen as a tool to direct provision. In land-based gambling, taxation has been used as a policy tool to deter inter-jurisdictional competition [47]. The same policy measure can be applied to online gambling. Online gambling markets are growing rapidly. In Europe, estimates suggest that the gross gambling revenue of the online market is expected to reach over 36 billion euros by 2026 [59]. A non-negligible part of this market is controlled by so-called offshore gambling. Estimating the size of the offshore market is difficult, but data from H2 Gambling Capital [15] has estimated the offshore market at around 13 % of the total European online market.

Offshore gambling is usually provided from low-tax offshore jurisdictions, such as Malta or Gibraltar. Many point-of-consumption jurisdictions therefore seek to balance revenue collection and competitive taxation to direct, or channel, consumers away from offshore markets [27]. Channelling refers to directing online gambling toward the regulated markets, using different measures such as blocking or making regulated offer more attractive to consumers and providers [35]. Many European jurisdictions highlight channelling as one of their key policy rationales for gambling, alongside consumer protection, crime prevention, and revenue interests [32]. As offshore gambling is connected to elevated levels of problem gambling severity [25, 27], channelling consumption has also been expected to promote harm reduction by directing consumers to offers that abide by local ‘responsibility’ and integrity standards [3, 33]. Channelling is often measured via so-called channelling rate which refers to the share of the onshore market of the overall market.

Alongside regulators, gambling industry stakeholders participate in the debate on optimal tax levels. For example, in the UK, industry representatives have argued that lower taxation would improve their competitive advantage over offshore provision and could therefore be used as a tool to channel the gambling monies towards the regulated market [61]. A 2015 report produced by Copenhagen Economics [13] and commissioned by BOS (Branchföreninengen för Onlinespel, Swedish Trade Association of Online Gambling, representing gambling companies and game developers that target Swedish market), suggests that a higher tax rate may be connected to a lower channelling rate. According to the report, an optimal channelling rate would be achieved with a tax rate of 15–20 percent on GGR, whereas tax rates above 20 percent on GGR would lead to lower channelling rates and lower overall tax revenues. This is because consumers would choose operators outside the regulated system. Another report, funded by the Danish online gambling industry [28, 29] similarly compared channelling rates and taxation levels in six European countries, concluding that whilst markets are not directly comparable, countries with a higher tax rate tend to have a lower channelling rate. Both studies included a comparative setting across six countries.

### This study

The current study produces more up-to-date evidence based on two cross-sections (2018; 2021) derived from a larger number of European countries (N = 29). Our aim is to investigate whether there is a negative correlation between channelling rate and tax rate. In the following, we describe the methods and data, present the main results, and discuss the implications of the study from the perspective of evidence-based policy, harm reduction, and public health objectives.

## Methods and data

We use data on tax rates and market shares of onshore and offshore online casino-type gambling and betting, as well as data on whether policies to block offshore gambling are in place. Data were collected from 29 European countries (EU-27, United Kingdom (UK), and Norway) and consist of two datapoints: 2018 and 2021. The data were collected from two databases that track regulatory and market developments in the gambling industry. Legal provisions on tax rates and blocking were retrieved from Vixio Gambling Compliance (Vixio GC), and channelling rate data were retrieved from H2 Gambling Capital (H2GC). Both data services were accessible to members of the research team via license.

### Channelling rate data

The data on online casino and online betting market shares (onshore and offshore) are based on estimates provided by H2 Gambling Capital [30]. H2 Gambling Capital is a UK-based data and market intelligence company specialising in gambling. The H2 estimates for onshore gambling are based on reported national figures. Estimates for offshore gambling are based on a model developed by H2 that consists of published company data, other assessment of the supply, in-house tracking, and contacts with private organisations, subscribers, providers as well as other industry analysts (the model is described for example in [29]). While these are only estimates, H2 figures are widely used by governmental actors as the best available source of information about gambling markets.

The dataset on estimated market value of onshore and offshore gambling provision is divided between online casino and online betting products in the 29 European countries for 2015, 2018 and 2021. The channelling rate (share of onshore gambling of the total online market) was calculated based on these for each country, and for the three time points. We included 2015 as this was also the year employed in a prior analysis [13]. Years 2018 and 2021 were added to study the market evolution at three-year intervals. The year 2021 is also expected to reflect the post-pandemic shift in the gambling sector, with likely increases in online gambling in comparison to 2018. 2021 data were also the most recent available data for all countries during data collection.

There are some differences across European countries in terms of which online gambling products are legal. However, the spread of licensing regimes and concentration of supplier markets in Europe have worked to unify product selections across countries and operators. The main outlier in the European market is France where only online poker is legal within the casino product category. In terms of offshore gambling, similar offers are available to residents of any European country.

### Tax data

The tax data are based on gambling taxation rates provided in Vixio GC country reports and news reporting. Up-to-date tax rates were collected on Vixio GC for 2021. We also included tax rates for 2018 by surveying the database for tax-related news articles for any changes in the included countries. For country-specific reasons (including existence of monopoly structures), it was not possible to include data on gambling taxation for all 29 countries in the initial sample. Moreover, unlike for channelling rates, tax figures from 2015 were not included, due to difficulties in obtaining reliable data from the sources available to us. Tax rates were collected separately for online casino and online betting, when these differed. Reported rates consist of a specific gambling tax, but in some cases (Netherlands, France) also of additional mandatory levies for earmarked purposes. These were summed together as a total tax rate.

Gambling companies can also pay corporate tax as well as license fees, but practices vary to a significant degree and could not be systematically assessed in this paper. Overall, both corporate tax and license fees constitute a minor share of the overall tax burden on gambling companies [37]. Moreover, corporate decisions on production levels depend on ‘marginal’ strategies [6]. Therefore, these taxes have been excluded from the analysis. In some countries, gambling winnings may also be subject to income. These have also been excluded as these are not common in Europe [21] and are unlikely to have a major effect on the interest of a company to establish in a particular jurisdiction.

Within the European Union, gambling is not subject to value added tax (VAT). The EU VAT directive (2006/122/EC) requires member states to exempt without a right of deduction of input VAT on ‘betting, lotteries and other forms of gambling, subject to the conditions and limitations laid down by each Member State (Article 135(1) (i)). Most gambling in Europe is therefore VAT-exempt. The European Commission has highlighted that the exemption for gambling is based on the practical difficulties rather than on a desire to make gambling more affordable for consumers [19].

### Blocking data

We also collected data on whether legal provisions existed for payment or website blocking in the 29 countries. Data were collected from Vixio Gambling Compliance country reports. The purpose of these data was to control for whether these could have effects on the results. Payment or website blocking may function as an obstacle to offshore gambling and may maintain higher channelling rates [16]. While it could not be established to what extent legal provisions on blocking translate to enforced policy across different European countries, the existence of blocking provisions may nevertheless deter companies from targeting certain markets with offshore provision. Blocking, whether enforced or not, may also function as an informative tool for consumers and providers. Blocking data were collected retrospectively in 2023 and may not be reflective of practices at the earlier (notably 2015 and 2018) datapoints.

### Methods

First, we provide a descriptive overview of the recent evolution of market channelling and taxation for online gambling products across Europe. Second, we produce a replication of previous empirical analyses [13, 28, 29] by introducing a multivariate regression model which assesses whether the extent of market channelling is correlated with taxation of online gambling. To guarantee comparability between empirical methods, we chose a standard ordinary least squares model (OLS), which closely matches the graphical scatterplots and linear interpolations presented in the reference studies [13, 28, 29]. We also introduce cross-sectional robustness checks: the usage of a variable measuring the presence in each country of systems for payment blocking and website blocking, the test of a quadratic relation between channelling and taxation, and further macro-economic controls. The usage of panel or first-difference methods is prevented by the rationale of our study (and its replication purposes), the shortness of our panel (only two time points) and lack of relevant covariates.

For each type of gambling product, we estimate the following Eq. 1, where *Ch* represents the country’s channelling ratio in, respectively, betting or casino games (henceforth *ChBe* and *ChCa*), *tax* their domestic taxation rate (*taxBe* and *taxCa*), the index *i* identifies the country and *t* the year of observation (*t* = 2018, 2021). *X* is a vector of controls, referring to the presence of country provisions on payment or website blocking (respectively, *pw* and *wb*) and further variables expressing macro-economic features and shocks which were found to impact on the gambling industry, such as GDP growth, population size, unemployment and inflation (e.g., [40, 50]). Unfortunately, the retrospective data collection did not allow us to create a specific vector for 2018, concerning payment or website blocking.

1 $$Ch_{it} = \alpha + \beta tax_{it} + \gamma X_{it} + \varepsilon_{it}$$

The OLS model presents heuristic limitations when used for inference, although it is an improvement over simple bivariate data interpolation. In our context, the estimated relation between channelling and taxation may suffer from a few econometric biases. Fiscal policies may not be random and independent, as assumed by the OLS model, and endogeneity and reverse causation may occur. For instance, some countries may set gambling taxation based on motivations external to the sector, but specific to the country (e.g., the amount of public debt or fiscal deficit). Similarly, the relative extent of the domestic and offshore markets (reflected into the dependent variable *Ch*) may condition gambling policies—including domestic taxation. Finally, domestic taxation is just one of the determinants of the equilibrium market price, in addition to other demand and supply factors. Therefore, we must interpret the OLS estimated relation as a mere statistical association, that does not support the causality claims explicitly posited in previous literature, which derives fiscal policy implications to maximise channelling or other target variables.

All analyses were performed with the software Stata 15, SE version.

## Results

### Taxation levels of gambling across Europe

Our analysis shows sizable differences in terms of how betting and online casino products are taxed. Table 1 shows descriptive statistics on the tax rates for those European countries for which data were available.

**Table 1:** Tax rates in Europe for online betting and casino, 2018 and 2021 – Descriptive statistics. *Source*: our computation on Vixio GC country reports and news reporting

Type of gambling	N	Mean	Standard deviation	Coefficient variation	Median	Min	Max
Tax Betting 2018 (GGR)	14	17.9	7.9	0.44	20.0	5.0	35.0
Tax Betting 2018 (R)	8	7.6	4.7	0.62	7.0	2.0	15.0
Tax Betting 2021 (GGR)	16	21.5	12.2	0.57	20.5	5.0	54.9
Tax Betting 2021 (R)	9	9.5	8.8	0.93	6.0	2.0	30.0
Tax Casino 2018 (GGR)	18	20.2	9.0	0.44	20.0	5.0	40.0
Tax Casino 2021 (GGR)	20	20.9	9.1	0.43	20.5	5.0	40.0

Descriptive statistics for online casino games taxed on turnover revenue are omitted because they refer to just one country in 2018 (France), and two countries in 2021 (France and Hungary)

Figures show that online betting products are taxed either at a gross gambling revenue (GGR) or a revenue (turnover) basis (R). Most countries adopted the GGR basis, both in 2018 (14 cases of 22) and 2021 (16 cases of 25). Online casino products are predominantly taxed at a GGR basis, except for poker that in a few countries is taxed at a R basis. Countries having state monopolies (Finland, Norway, Luxembourg) are excluded from this table for missing values. In these countries, profits are transferred to state using other configurations. Poland and Slovenia also operated partial monopolies and only taxed the competitive segment of the market. Table A1 in the Appendix provides fuller details on individual countries’ taxation choices.

Descriptive statistics of Table 1 yield two key findings. First, the level of taxation (expressed in percent values) ranges across European countries, for both products and periods. For products taxed at a GGR basis, average taxation starts from the 18% threshold, and grows to 21.5% by 2021. For R-based taxation, the initial reference average is around 8%, but also grows across time. Second, the average growth of taxation levels is accompanied by an increase of country heterogeneity. This is also reflected in the coefficient of variation and the Max values: the first generally augments from 2018 to 2021; the second ones tend to double in the same period. Table A1 in appendix shows that there is a certain persistence of taxation around the Min values, for a small set of countries, such as Malta and Estonia (5% on GGR for both products).

In terms of blocking policies, 18/29 countries reported payment blocking and 20/29 countries reported website blocking. Only four countries (Austria, Ireland, Malta, and the UK) had no blocking policies (see Table A1 in supplementary material).

### Channelling rates across Europe

Table 2 provides descriptive statistics on our data on channelling rates in Europe for 2015, 2018, and 2021 (N = 29). Overall, the results show that channelling rates are increasing in Europe. For online betting, the European mean for channelling has increased from 42.2 percent in 2015 to 58 percent in 2018 and 64.9 percent in 2021. For online casino products, the mean of the European channelling rate has increased from an average of 24.2 percent in 2015 to 40.5 percent in 2018 and 55.1 percent in 2021. Similarly, the median shows that the central tendency of the distribution has continuously grown from 2015 to 2021 for both product categories. The Max value shows that in all markets and periods, frontrunners for channelling have a predominantly domestic online market (channelling close to 100%). Finally, contrary to the diverging patterns of taxation, the European increase in channelling has occurred within a pattern of country homogenisation (the coefficient of variation monotonically diminished for all products and periods).

**Table 2:** Channelling in Europe for online betting and casino, 2015, 2018, and 2021 – Descriptive statistics. *Source*: our computation on H2 GC (release 2022) data

Type of gambling	N	Mean	Standard deviation	Coefficient variation	Median	Min	Max
Ch Betting 2015	29	42.2	30.3	0.72	43.0	0.0	97.6
Ch Betting 2018	29	58.0	30.3	0.52	59.2	0.0	98.0
Ch Betting 2021	29	64.9	31.5	0.49	76.0	0.0	98.4
Ch Casino 2015	29	24.2	28.6	1.18	8.1	0.0	95.0
Ch Casino 2018	29	40.5	33.5	0.83	43.0	0.0	95.6
Ch Casino 2021	29	55.1	35.0	0.63	62.4	0.0	96.1

Ch, for all products and years, is calculated as the ratio of the relevant onshore consumption on the total (onshore + offshore) consumption

Table 3 presents the matrix of pairwise correlations of the channelling ratios for both products (abbreviated as *ChBe*- and *ChCa*-), across countries and years. The autocorrelation coefficients are all significant, and the coefficient size for any [*t, t-1*] pair is higher than for [*t, t-2*] pairs, and mostly fluctuates around the 0.8 threshold: this is a sign of growth persistence at the country-level. These results provide further evidence on the occurrence of a general trajectory of cumulative growth in the European markets.

**Table 3:** Pairwise correlation analysis of channelling rates. *Source*: our computation on H2 GC (release 2022) data

	ChBe15	ChBe18	ChBe21	ChCa15	ChCa18	ChCa21
ChBe15	1					
ChBe18	0.7804*	1				
ChBe21	0.5994*	0.8716*	1			
ChCa15	0.6412*	0.4651*	0.2479	1		
ChCa18	0.4703*	0.5777*	0.3918*	0.7550*	1	
ChCa21	0.4415*	0.6547*	0.5627*	0.5425*	0.8565*	1

*Pairwise correlations significant at *p* = 5%. N = 29

**Fig. 1:**
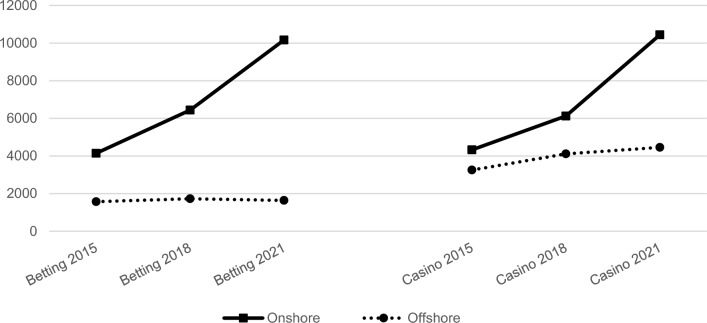
Evolution of online gambling consumption in Europe: onshore vs offshore components (constant Mil. $). *Source*: our computation on H2 GC (release 2022) data. Absolute values deflated at 2015 constant terms (Mil. $), using the specific national Harmonised Indexes of Consumer Price (Eurostat)

Figure 1 presents the results of the H2 GC estimates of the volumes of online gambling consumption for our European sample, across 2015–21. It shows that the onshore component experienced a relevant growth for both products (increasing by more than 140% in 2015–21), whereas the offshore component increased by only 4% (betting) and 37% (casino)—when expressed in real (deflated) terms. The increasing channelling rate indicates that at a European level the gambling industry is growing particularly within the regulated market. The offshore market is growing at a slower pace, with proportionate market share shrinking at the European level. Growth of regulated markets is occurring in most European markets, attenuating cross-country differences.

### Relationship between tax rate and channelling

Previous reports [13, 28, 29] have suggested a possibly negative correlation between channelling rate and taxation rate using simple bivariate scatterplots between channelling and taxation and (linear) interpolations, alongside an expected causality link going from taxation to channelling. We produced a similar analysis using the simple OLS model presented in Eq. 1, which closely matches the above empirical analyses and enables direct comparability of results.

Table 4 presents the estimates of the different versions of the OLS model for betting (version 1–3 for 2018, version 4–6 for 2021). In detail, columns 1 and 4 correspond to a linear model without blocking controls, columns 2 and 5 include the controls, and columns 3 and 6 consider a similar OLS model featuring also a quadratic term (*taxBeYEARsq*), with controls. In fact, a statistically significant relation between channelling and taxation might exist but be non-linear; and this needs to be specifically tested. Basically, columns 3 and 6 correspond to the generic model represented by Eq. 2 (henceforth, ‘quadratic version of the OLS’), where *tax*^*2*^ is the square of the tax rate and ${\beta }_{2}$ its estimated coefficient (we recall that a negative value would depict a relation between channelling and taxation shaped as an inverse U):

2 $$Ch_{it} = \alpha + \beta_{1} tax_{it} + \beta_{2} tax_{it}^{2} + \gamma X_{it} + \varepsilon_{it}$$

**Table 4:** OLS regressions on channelling and taxation, online betting in 2018 and 2021

VARIABLES	(1)	(2)	(3)	(4)	(5)	(6)
	ChBe18	ChBe18	ChBe18	ChBe21	ChBe21	ChBe21
taxBe18	3.133***	3.137**	8.777***			
	(0.930)	(1.013)	(1.232)			
taxBe18sq			− 15.41***			
			(2.582)			
wb		0.0166	0.0544		0.155	0.187
		(0.118)	(0.111)		(0.230)	(0.244)
pb		− 0.0162	− 0.0587		− 0.0276	− 0.112
		(0.137)	(0.100)		(0.248)	(0.198)
taxBe21				1.143*	1.054	4.480**
				(0.595)	(0.607)	(1.689)
taxBe21sq						− 5.981**
						(2.494)
constant	0.115	0.115	− 0.305**	0.471***	0.406	0.0764
	(0.160)	(0.215)	(0.125)	(0.153)	(0.271)	(0.250)
N	14	14	14	16	16	16
R-squared	0.700	0.701	0.904	0.204	0.255	0.434
Prob > F	0.006	0.066	0.000	0.075	0.258	0.088
Max taxBe_year			0.285***			0.374***
			(0.011)			(.046)

Robust standard errors in parentheses. *** p < 0.01, ** p < 0.05, * p < 0.1

Unfortunately, the different country taxation systems prevented us from exploiting the full dataset: In fact, there is not documented way (nor literature guidance) to estimate a reliable correspondence between taxation at a gross gambling revenue (GGR) and taxation at a revenue (turnover) basis (R), since gambling values chains vary depending on country, company, product portfolios and time. Any reconstruction attempt would introduce a non-negligible bias in the recomputed *taxBeYEAR and taxGaYEAR* core variables. Therefore, Table 4 only exploits the larger sub-datasets (N = 14 and N = 16), which corresponds to the countries using the more frequent system of taxation for betting—that on GGR. Results show that the 2018 model (versions 1–3) passes the F test (explanatory power is generally superior to that of the basic model featuring the constant); equally, the R-squared assumes values within the conventional range for cross-sections. Instead, the 2021 model is only marginally significant for version 4 and 6 (the F test passes only at 10% level), and ‘totally’ insignificant in 5. Therefore, versions 1–2 suggest that in 2018 there is a positive and significant linear relation between taxation and channelling, whereas version 3 adds that the significant relation is a quadratic one, depicting an inverse U-shaped relation between the two variables: for lower levels of taxation, increasing taxes is compatible with higher channelling, up to a maximum; afterwards, higher taxation would be associated to lower (diminishing) channelling. Finally, countries having a payment or website blocking policy do not report any statistically significant influence on channelling associated to it.

Table 5 performs the same analysis for casino games, for both periods: 2018 (versions 1–3), and 2021 (versions 4–6). This model does not reach the minimal significance level (as showed by the F test and the poor value of the R-squared) in any period: a model featuring just the constant is superior to our linear or quadratic versions. This means that our data on online casino gambling show no trace of statistical association between taxation and channelling. Instead, these appear to have evolved along disjoint paths.

**Table 5:** OLS regressions on channelling and taxation, online casino in 2018 and 2021

Variables	(1)	(2)	(3)	(4)	(5)	(6)
	ChGa18	ChGa18	ChGa18	ChGa21	ChGa21	ChGa21
taxCa18	0.081	− 0.072	2.234			
	(0.782)	(0.842)	(3.476)			
taxCa18sq			− 5.431			
			(7.016)			
wb		0.171	0.127		0.155	0.105
		(0.205)	(0.246)		(0.167)	(0.166)
pb		− 0.106	− 0.123		0.033	0.017
		(0.196)	(0.188)		(0.143)	(0.132)
taxCa21				0.0314	0.0383	2.823
				(0.889)	(0.782)	(3.402)
taxCa21sq						− 6.571
						(6.817)
Constant	0.540**	0.523	0.365	0.683***	0.550*	0.353
	(0.198)	(0.353)	(0.424)	(0.222)	(0.314)	(0.416)
N	18	18	18	20	20	20
R-squared	0.001	0.052	0.085	0.000	0.069	0.116
Prob > F	0.919	0.832	0.563	0.972	0.802	0.701

Robust standard errors in parentheses. ***p < 0.01, **p < 0.05, *p < 0.1

The model is limited by the scarcity of the available controls, which stems from the poor involvement of official statistics in the coverage of the gambling sector. To account for these issues, we use macroeconomic controls, which we present in the last battery of regressions as robustness checks. For parsimony, we only present those model specifications reaching minimal significance levels. The non-significant results of the specifications for casino gambling are available from the authors upon request.

Table 6 presents the robustness checks for betting, for 2018. Checks consist in the inclusion of macro-economic controls (GDP variation, unemployment rate, country population, and inflation rate—description and statistics on these regressors are reported in Table A2). These covariates were found to display an impact on gambling markets in the literature [40, 50]. Results confirm the significant correlation between channelling and taxation uncovered in Table 4. The latter continues to be verified in the linear (1–2) and in the quadratic versions (3–4) of the model, with the quadratic version receiving more support (according to the R-squared and F test). Conversely, the inclusion of these macroeconomic controls in the 2021 confirms the evidence of Table 4 (regarding its versions 4–6): the F-test remains barely significant, and the previous marginally significant correlation between channelling and taxation disappears, both in the linear and quadratic versions (results available from the authors).

**Table 6:** OLS regressions on channelling and taxation, online betting in 2018: robustness checks

VARIABLES	(1)	(2)	(3)	(4)
	ChBe18	ChBe18	ChBe18	ChBe18
taxBe18	3.327***	3.210***	7.759***	7.312***
	(0.600)	(0.546)	(1.016)	(1.539)
rGDPvar18	− 0.0348	− 0.0194	− 0.0182	− 0.0121
	(0.0278)	(0.0394)	(0.0188)	(0.0279)
unemp18	− 0.0211*	− 0.0208**	− 0.00626	− 0.00727
	(0.0108)	(0.00832)	(0.00435)	(0.00495)
popsh18		0.0124		0.00623
		(0.00810)		(0.00801)
infl18		− 0.00200		0.00110
		(0.0561)		(0.0329)
taxBe18sq			− 12.83***	− 11.70**
			(2.604)	(4.140)
Constant	0.355*	0.277	− 0.120	− 0.122
	(0.181)	(0.206)	(0.152)	(0.161)
N	14	14	14	14
R`squared	0.810	0.842	0.908	0.915
Prob > F	0.001	0.000	0.000	0.000
Max taxBe_year			0.302***	0.312***
			(0.026)	(0.047)

Robust standard errors in parentheses. ***p < 0.01, **p < 0.05, *p < 0.1

Finally, sticking to the quadratic version of the OLS model (Eq. 2), simple calculations yield the maximum ‘bearable’ level of taxation (*Max taxBe_year* in Tables 4 and 6). This value corresponds to the mathematical point of maximum of the parabolic relationship (shaped like an inverted U) linking channelling with taxation. At this point, channelling reaches its maximum attainable value. Of course, the parameter *Max taxBe_year* is presented following an ‘a contrario’ logic, and cannot be granted a causal interpretation, given the econometric biases of the OLS model previously illustrated. Results show that the maximum levels of taxation we calculated are much higher than the optimal ones previously suggested at around 15% (cf. Copenhagen [13]). Based on our data, *Max taxBe_year* lies in the 28–37% range, across the various model versions.

## Discussion

This study has mapped the tax levels, channelling rates, and their correlation across 29 European countries. Our results have shown that, first, there is important divergence in approaches to gambling taxation across European countries and across product groups. Our analysis included figures on online casino and online betting products. We found that while online casino products are predominantly taxed at a GGR basis, the approaches to taxing betting products are more evenly split between GGR-based taxation and turnover-based taxation. Adopted tax rates also varied significantly, with some jurisdictions (including Malta) offering very advantageous tax rates (5%/GGR), while other countries taxed GGR at rates over 50%.

Some of this divergence in taxation can be explained by national differences in terms of approaches to taxation more generally [20]. The European Commission has not harmonised its approach to gambling services. Member states have the power to decide on the most appropriate way to tax gambling. However, the European Commission has questioned national approaches to the taxation of online gambling, particularly when this has been much inferior to taxation levels of land-based gambling. For instance, in a formal investigation on Danish taxation practices for the land-based (45%) and online operators (20%), the Commission concluded that while this can be a seen as state aid, this ‘state aid’ was proportional for its purpose, i.e., channelling consumption from the offshore market [43].

In addition, European jurisdictions may differ in how they approach the balance between tax revenue needs and industry lobbying for advantageous taxation. For example, Lycka [38] has argued that gambling operators tend to prefer taxation on gross gambling revenue (GGR) over turnover-based taxation, as the latter is less profitable for them [38]. However, from a harm prevention perspective, turnover-based taxation could be more effective in preventing the offering of most harmful forms of gambling. As gambling demand is largely inelastic for those experiencing problems, this would allow a preventive approach by making the most harmful gambling products less attractive to consumers and to providers.

Second, our results showed that channelling rates have increased in Europe across our period of observation (2015–2021). Although increases have been observed in some countries, the overall trend has been that of decreasing offshore market shares. This result is supported by other reports [15]. However, the decrease of the offshore market shares is mostly attributable to the absolute growth of the onshore market (more than 140% in 2015–21). The growth of the offshore market, in real terms, was only 4% for betting and 37% for casino products. This evolution therefore seems to be structural rather than conjunctural. Channelling rate, although often used in policy evaluations, is not a comprehensive indicator of market success as it is sensitive to changes in both onshore and offshore markets. Further studies should focus more thoroughly on whether channelling rates are even a good measure of regulatory success.

In addition to on-going digitalisation and relaxation of pandemic restrictions in the regulated European market [4], the growth of onshore gambling may be explained by improved control, including website and payment blocking schemes (cf. [16]) and other enforcement. Furthermore, the introduction of licensing systems in many European jurisdictions has brought many formerly offshore providers under the umbrella of the regulated market [3]. In 2015, many countries had not regulated online gambling, at least in some sectors, and all gambling within that sector took place in the offshore market—this resulted in channelling rates of zero percent for some countries.

Third and finally, our analysis has shown that taxation rates do not appear to be negatively correlated with channelling rates within Europe, as found by Copenhagen Economics [13] and H2 [29]. Instead, our results suggest that in some cases, higher tax rates may even be associated with more effective channelling, both in a linear and in a quadratic shape. Our inferential results call for additional econometric tests based on larger datasets. However, our study represents a step forward with respect to previous analyses that have been based on descriptive statistics and on smaller groups of countries. The difference between our results and earlier reports can be a result of different years of observation, sampling and usage of controls. Earlier reports only included six jurisdictions, did not differentiate between casino and betting types of gambling, and did not perform regressions controlling for other covariates.

Furthermore, a more case-specific reading of our data suggests that even countries with a comparatively high tax rate on gambling (France, Germany) can have a high channelling rate. Increases in tax rate have also not been reflected as reduced channelling rates. For example, in Denmark, tax level was increased from 20%/GGR to 28%/GGR in 2021. The channelling rate nevertheless remained stable, and even increased for online betting across 2018–2021. This finding was also contrary to a report commissioned by the Danish gambling industry in 2020, suggesting that the tax increase would result in a reduction of channelling rate from 84 to 76% [28, 29].

Gambling policy is often based on assumptions that can still be used by regulators or industry representatives. Wardle et al. [61] have called narratives advancing a threat of black-market provision of gambling a form of ‘regulatory resistance’. Regulatory resistance is characterised by industry-led argumentation, often using industry-generated evidence, and arguments that reduced regulation can ‘protect’ the industry from the black market. When independent, empirical evidence is not available, or is scarce, this type of argumentation can be very influential in policymaking, including promoting advantageous fiscal policy [14, 61]. Empirical research into the effects of tax levels or other restrictive policies on gambling are needed to base regulatory approaches on best possible evidence.

This study has limitations. First, existing sources do not offer longitudinal evidence (a panel) on the key variables we studied, but just a few yearly waves. More generally, access to data continues to be a problem in the gambling research field. The data used for this study were accessed under license from the gambling sector intelligence services. The data on offshore and onshore markets provided by H2 Gambling Capital are based on estimates produced from various data sources, but the company does not publicise the exact basis of the calculations. Channelling rates also only reflect the relative shares of onshore and offshore markets and are therefore sensitive to a variety of changes within both. In addition, while these estimates are widely used in the field, they are impossible to ascertain due to the highly heterogenous and constantly changing nature of offshore gambling provision. Similarly, the data on gambling taxation and blocking policies retrieved from Vixio Gambling Compliance is subject to the tracking conducted by this data provider. For instance, it is possible that some tax policy changes were not reported in the database and are therefore missing from our dataset.

The important heterogeneity of taxation practices in Europe complicated our analyses, reduced the sample size, and resulted mostly in statistically non-significant results. The small cross-sectional samples we used do not cater for the asymptotic properties of the OLS estimator. Some countries were excluded from our analyses as they operate monopolistic regimes or had strongly varying taxation rates depending, for example, on company size. Similarly, the taxation of online betting was roughly divided into GGR and turnover-based models, making it necessary to conduct these analyses separately. There was also variance in terms of how unauthorised gambling is prevented via means of blocking. Although most countries had some provisions for blocking, these are not always enforced due to resource constraints or other political reasons. This could explain the statistical insignificance of the coefficients of the blocking covariates (*wb* and *pb*).

It was also not possible to address causality claims with this model, given the simultaneity and endogeneity of the core relation. Therefore, any correlation must be interpreted as simple association, and not as causality.

Our best attempt to estimate the maximum ‘bearable’ rate of taxation produced an estimate lying around the 30% threshold, which is nearly the double of what was predicated by previous studies (e.g., 15% [13]). This result shows the difficulty in producing such recommendations, as the optimal tax rate is likely to vary significantly depending on contextual factors. Producing solid recommendations on optimal taxation remains complicated because a high enough tax rate on gambling may hinder market growth and deter some operators, but it may also translate to lower return rates to players and therefore higher losses. Furthermore, significant state revenue from gambling taxes involves a risk of state governments and other beneficiaries becoming dependent on these revenue streams. This can have negative impacts of policy, as states may become complicit in the promotion of gambling [1, 11, 44–46, 56]. A more sustainable solution may be to continue the already existing trend of taxing online gambling based on point-of-consumption from point-of-sale [59], as well as collaboration within Europe to enforce this.

## Conclusion

The results of this study have shown how important evidence-based policy is in reducing and preventing the negative consequences of harmful online gambling. Taxation of gambling is unlikely to significantly direct consumption, but it can be an important tool in preventing some gambling harms by making the provision of certain product categories less attractive to providers. Taxation may be one aspect in the on-going work of regulators to direct consumption away from offshore markets, but it is unlike to be enough as a stand-alone measure. From a public health perspective, harm prevention and harm reduction is more effective if total consumption is reduced [54], rather than just shifted from one market segment to another. Higher taxation of gambling may play a part in making gambling less attractive to consumers and operators. The results of our study suggest that higher taxation is unlikely to drive consumption to offshore markets.

## Supplementary Information

Supplementary Material 1

## Data Availability

Data used in this study are available via license from Vixio Gambling Compliance and H2 Gambling Capital. Restrictions apply to the availability of these data, which were used under license for the current study, and so are not publicly available. Data are however available from the authors upon reasonable request and with permission of Vixio and H2.

## Abbreviations

BOS: Branchföreninengen för onlinespel
EU: European Union
GDP: Gross domestic product
GGR: Gross gambling revenue
H2GC: H2 Gambling Capital
OLS: Ordinary least squares
R: Total revenue
UK: United Kingdom
VAT: Value added tax
Vixio GC: Vixio Gambling Compliance
