# Supplementary material for: Channelling and taxation in European online gambling markets: evolution and policy implications
Source: Harm Reduct J. 2025 Jan 3;22:1. doi: 10.1186/s12954-024-01145-0 (PMC11699665; doi:10.1186/s12954-024-01145-0)
Supplement: Supplementary file 1 — Supplementary Material 1 [file 12954_2024_1145_MOESM1_ESM.docx]

**Supplementary materials:**

Table A1: Gambling tax levels and blocking policies in 29 European countries (2021)

|  | Online casino | Online betting | Website blocking | Payment blocking |
| --- | --- | --- | --- | --- |
| Austria | 40 %/GGR | 2%/R | No | No |
| Belgium | 11%/GGR | 15%/GGR | No | Yes |
| Bulgaria | 20%/GGR | 15%/R | Yes | No |
| Croatia | 15%/GGR | 5%/R | Yes | No |
| Cyprus | NOT LEGAL | 13%/GGR | Yes | No |
| Czech Republic | 23%/GGR | 23%/GGR | Yes | Yes |
| Denmark | 28%/GGR | 28%/GGR | Yes | Yes |
| Estonia | 5%/GGR | 5%/GGR | Yes | Yes |
| Finland | MONOPOLY | MONOPOLY | No | Yes |
| France | 2%/R | 54,9%/GGR | Yes | Yes |
| Germany | Varies | 5.3%/R | Yes | Yes |
| Greece | 35%/GGR | 35%/GGR | Yes | Yes |
| Hungary | 15%/NGR | 30%/NGR | Yes | Yes |
| Ireland | NOT LEGAL | 2%/R | No | No |
| Italy | 25%/GGR | 24%/GGR | Yes | Yes |
| Latvia | 10%/GGR | 10%/GGR | Yes | Yes |
| Lithuania | 20%/GGR | 20%/GGR | Yes | Yes |
| Luxembourg | MONOPOLY | MONOPOLY | No | No |
| Malta | 5%GGR | 5%/GGR | No | No |
| Netherlands | 30.95%/GGR | 30.95%/GGR | No | Yes |
| Norway | MONOPOLY | MONOPOLY | No | Yes |
| Poland | MONOPOLY | 12%/R | No | Yes |
| Portugal | 25%/GGR | 8%/R | Yes | No |
| Romania | 21%/GGR | 21%/GGR | Yes | Yes |
| Slovakia | 27%/GGR | 6%/R | Yes | Yes |
| Slovenia | 18%/GGR | MONOPOLY | Yes | Yes |
| Spain | 20%/GGR | 20%/GGR | Yes | Yes |
| Sweden | 18%/GGR | 18%/GGR | No | Yes |
| United Kingdom | 21%/GGR | 21%/GGR | No | No |

Source: our computation on Vixio GC country reports and news reporting

Table A2: Descriptive statistics on further regressors

| **Variable** | **Definition** | **N** | **Mean** | **Standard Deviation** | **Coefficient Variation** | **Median** | **Min** | **Max** |
| --- | --- | --- | --- | --- | --- | --- | --- | --- |
| **wb** | Presence of web blocking | 14 | 0,7 | 0,5 | 0,66 | 1 | 0 | 1 |
| **pb** | Presence of payment blocking | 14 | 0,8 | 0,4 | 0,54 | 1 | 0 | 1 |
| **rGDPvar18** | Annual variation of real GDP | 14 | 3,3 | 2,0 | 0,59 | 2,8 | 0,9 | 7,4 |
| **unemp18** | Unemployment rate (on labour force) | 14 | 7,6 | 4,7 | 0,62 | 6,1 | 2,2 | 19,7 |
| **popsh18** | Country share of the EU-27 population | 14 | 4,0 | 5,1 | 1,28 | 2,4 | 0,1 | 14,9 |
| **infl18** | Inflation rate | 14 | 2,0 | 1,0 | 0,49 | 2,0 | 0,7 | 4,1 |
|  |  |  |  |  |  |  |  |  |

Source: our computation on: 1) Vixio GC country reports and news reporting for variables *wb* and *pb*; Eurostat data centre (<https://ec.europa.eu/eurostat/web/main/data/database>) for all the remaining variables.
